# Fixed point results for generalized rational type $\alpha$ -admissible contractive mappings in the setting of partially ordered b-metric spaces

**DOI:** 10.1186/s13104-022-06122-z

**Published:** 2022-07-07

**Authors:** Kedir Husen Haji, Kidane Koyas Tola, Mustefa Abduletif Mamud

**Affiliations:** Department of Mathematics, Jimma University, Jimma, Ethiopia

**Keywords:** Fixed point, Partially ordered b-metric space, Generalized rational type $\alpha$ -admissible contractive mappings

## Abstract

**Objectives:**

In this paper we introduce fixed point theorems for generalized rational type $\alpha$ -admissible contractive mappings in partially ordered b-metric spaces and prove the existence and uniqueness of fixed points for self-mappings satisfying the established theorems. Finally, we provide examples in support of our main findings in the setting of partially ordered b-metric spaces.

**Result:**

New fixed point results have been obtained for generalized rational type $\alpha$ -admissible contractive mappings in the setting of partially ordered b-metric space and we applied one of our results to determine a solution to an integral equation.

## Introduction

Banach contraction principle [2] is one of the most useful results in nonlinear analysis. It ensures the existence and uniqueness of the fixed points of nonlinear operators satisfying strict contractive conditions. It also shows that the fixed point can be approximated by means of a Picard iterations. Due to its application in mathematics and other related fields of study the Banach contraction principle has been generalized in many directions. One of the generalizations of Banach fixed point theorem is the one given in the setting of partially ordered metric spaces which was initiated by Wolk [3]. After that Ran and Reurings [4] introduced fixed point results in the setting of partially metric spaces. There after Nieto and RodriguezLopez [5] extended the works of Ran and Reurings for non-decreasing mappings and applied their results to determine a solution of certain differential equation. For more fixed point results in partially ordered metric and partially ordered b-metric spaces readers may refer to [6–11] and the references therein. Recently, in 2020, Seshagiri Rao and Kalyani [1] defined generalized rational type contraction mappings and studied fixed point theorems for the class of mappings introduced in the setting of partially ordered metric spaces.

Inspired and motivated by the works Seshagiri Rao and Kalyani [1] in this paper we introduce generalized rational type $\alpha$ -admissible contractive mappings and study fixed point results in the setting of partially ordered b-metric spaces.

## Preliminaries

In this section, we recall some basic definitions and results which we use in the sequel.

### Notation 1

In this paper we denote:

1. $$R^{+} = [0,\infty );$$
2. *R* is the set of real numbers;
3. *N* is the set of natural numbers.

### Definition 1

[12] Let *X* be a nonempty set and $s\ge 1$ be a given real number. A function $d:X\times X \rightarrow R^{+}$ is said to be a b-metric if and only if for all $x,y,z\in X$, the following conditions are satisfied:

(i) $d(x,y) = 0$ if and only if $x = y$;
(ii) $$d(x,y) = d(y,x)$$;
(iii) $$d(x,z)\le s[d(x,y)+d(y,z)]$$.

The pair (*X*, *d*) is called a b-metric space.

It should be noted that, the class of b-metric spaces is effectively larger than that of metric spaces, since a metric is a b-metric with $s=1$.

But in general, the converse is not true.

### Example 1

[13] Let $X = R$ and $d:X\times X \rightarrow R^{+}$ be given by $d(x,y) = |x-y|^{2}$ for $x,y\in X$, then *d* is a b-metric on *X* with $s = 2$ but it is not a metric on *X* since for $x = 2,y = 4$ and $z = 6$, we have

$$\begin{aligned} d(2,6)=16\nleq 8=4+4= d(2,4)+d(4,6). \end{aligned}$$

Hence, the triangle inequality for a metric does not hold.

### Definition 2

[14] Let $(X,\preceq )$ be a partially ordered set. A sequence $\{x_n\}$ in *X* is said to be non-decreasing with respect to $\preceq$ if $x_n \preceq x_{n+1}$ for all $n\in N.$

### Definition 3

[14] Let $(X,\preceq )$ be a partially ordered set and $T:X\rightarrow X$ be a mapping then,

**(i)**: elements of $x,y\in X$ are said to be comparable if $x\preceq y$ or $y\preceq x$ holds.
**(ii)**: a nonempty set *X* is called well-ordered set, if every two elements of it are comparable.
**(iii)**: *T* is said to be monotone non-decreasing with respect to $\preceq$ if for all $x,y\in X,$ $x\preceq y$ implies $Tx\preceq Ty$.
**(iv)**: *T* is said to be monotone non-increasing with with respect to $\preceq$ if for all $x,y\in X,$ $x\preceq y$ implies $Tx\succeq Ty$.

### Theorem 1

[15] *Let* (*X*, *d*) *be a complete metric space and suppose that there exist* $\alpha ,\beta \ge 0$ *with* $\alpha +\beta <1$ *and* $T:X\rightarrow X$ *satisfying the contraction condition:*

$$d(Tx,Ty)\le \alpha \frac{d(y,Ty)[1+d(x,Tx)]}{1+d(x,y)}+\beta d(x,y)$$ *for all* $$x,y\in X$$.

*Then* *T* *has a unique fixed point.*

### Theorem 2

[16] *Let* $(X,\preceq )$ *be a partially ordered set and suppose that there exists a metric* *d* *on* *X* *such that* (*X*, *d*) *is a complete metric space. Suppose that* *T* *is a continuous, monotone non-decreasing self- mapping on* *X* *and* $d(Tx,Ty)\le \alpha \frac{d(x,Tx)d(y,Ty)}{d(x,y)+d(x,Ty)+d(y,Tx))}+\beta d(x,y)$ *for all* $x,y\in X$*, with* $x\succeq y$ *and for some* $\alpha ,\beta \in [0,1)$ *with* $\alpha +\beta <1$*, there exists* $x_0\in X$ *with* $x_0\preceq Tx_0$.

*Then* *T* *has a fixed point.*

### Definition 4

[17] Let *X* be a nonempty set, $T :X \rightarrow X$ and $\alpha :X \times X \rightarrow R^{+}$, we say that $\ T$ is an $\alpha$-admissible mapping if $\alpha (x,y) \ge 1$ implies $\alpha (Tx,Ty) \ge 1$, for all $x,y \in X$.

### Definition 5

[18] Let $\ (X,d)$ be a b-metric space, $x\in X$ and $\{x_n\}$ be a sequence in *X*. Then we say that:

**(i)**: $\{x_n\}$ b-converges to *x* if $d(x_n,x)\rightarrow 0$ as $n\rightarrow \infty$.
**(ii)**: $\{x_n\}$ is a b-Cauchy sequence if $d(x_n,x_m)\rightarrow 0$ as $n,m\rightarrow \infty$.
**(iii)**: (*X*, *d*) is b-complete if every b-Cauchy sequence is b-convergent in *X*.

### Definition 6

[19] Let (*X*, *d*) be a *b*-metric space with the coefficient $s \ge 1$ and $T:X\rightarrow X$ be a given mapping. We say that T is continuous at $x_{o} \in X$ if and only if for every sequence $\{x_{n}\} \text { in } X,$ we have ${x_{n}}\rightarrow x_{o}$ as $n\rightarrow \infty ,$ then $Tx_{n} \rightarrow Tx_{o}$ as $n\rightarrow \infty .$

If *T* is continuous at each point $x_{0} \in X$, then we say that *T* is continuous on *X*. In general, a *b*-metric is not necessarily continuous.

### Example 2

Let $X= N U \{\infty \} .$ Define a mapping $d : X \times X \rightarrow R^{+}$ as follows:

$$\begin{aligned} d(m,n)= {\left\{ \begin{array}{ll} 0&{}\quad \text {if } m=n; \\ \left| \frac{1}{m}-\frac{1}{n}\right| &{}\quad \text {if one of }m\text { and }n\text { is even and the other even or }\infty ;\\ 5&{} \quad \text {if one of }m\text { and }n\text { is odd and the other is odd or }\infty ;\\ 4&{}\quad \text {if others,} \end{array}\right. } \end{aligned}$$

$$d(m,p)\le \frac{5}{4} [d(m,n)+d(n,p)] \ for \ all \ m,n,p\in X.$$

Then (*X*, *d*) is a *b*-metric space with $s =\frac{5}{4}$.

Choose $x_{n} = 2n$ for each $n\in N$. Then

$$\begin{aligned} d(x_{n},\infty ) =d(2n,\infty ) = \frac{1 }{2n}\rightarrow 0 \ as \ n\rightarrow \infty \end{aligned}$$

that is, $$x_{n} \rightarrow \infty \ as \ n\rightarrow \infty$$.

But,

$$\begin{aligned} \lim _{n\rightarrow \infty } d(x_{n},1) = 4\ne 5 = d(\infty ,1). \end{aligned}$$

Hence *d* is not continuous.

### Definition 7

[7] Let (*X*, *d*) be a complete metric space and $(X,\preceq )$ is partial ordered set. Then $(X,d,\preceq )$ is called a complete partially ordered metric space.

## Main results

Now we define generalized rational type $\alpha$-admissible contractive mappings in the setting of partially ordered *b*-metric space and prove fixed point results for the mappings defined.

### Definition 8

Let $(X,d,\preceq )$ be a partially ordered *b*-metric space with coefficient $s\ge 1$, $\alpha :X\times X\rightarrow R^{+}$ and $T:X\rightarrow X,$ then *T* is said to be generalized rational type $\alpha$-admissible contractive mapping if there exit $a,b,c,e,f \in [0,1)$ with $as+(2s+s^{2})b+c+es+f<1$ and satisfies:

1 $$\begin{aligned} \begin{aligned} \alpha (x,y)d(Tx,Ty) \le {\left\{ \begin{array}{ll} ad(x,y)+\ b[d(x,Tx)+d(x,Ty)] +\ c\frac{d(y,Ty)+d(y,Tx)}{1+d(y,Ty)d(y,Tx)} \\ \quad +e\frac{d(x,Tx)d(x,Ty)+d(y,Tx)d(y,Ty)}{d(y,Tx)+d(x,Ty)} +f\frac{d(y,Ty)[1+d(x,Tx)]}{1+d(x,y)}&{}\quad \text {if }A\ne 0 \\ 0&{}\quad \text {if }A=0 \end{array}\right. } \end{aligned} \end{aligned}$$

for all $x,y\in X$ with $x\preceq y$ where $A=d(y,Tx)+d(x,Ty)$.

### Theorem 3

*Let* $(X,d,\preceq )$ *be a complete partially ordered b-metric space with coefficient* $s\ge 1$*,* $\alpha :X\times X\rightarrow R^{+}$ *and* $T:X\rightarrow X$ *satisfies the following conditions:*

**(i)**: *T* *is generalized rational type* $\alpha$-*admissible contractive mapping;*
**(ii)**: *there exists a point* $x_0\in X$ *such that* $x_0\preceq Tx_0$ *and* $\alpha (x_0,Tx_0)\ge 1$*;*
**(iii)**: *T* *is continuous and a non-decreasing mapping with regards to* $\preceq$*;*
**(iv)**: *T* *is an* $\alpha$-*admissible mapping;*

*Then* *T* *has a fixed point in* *X*.

### Proof

By **(ii)** there exists $x_0\in X$ such that $x_0\preceq Tx_0$ and $\alpha (x_0,Tx_0)\ge 1$. We define a sequence $\{x_n\}$ in *X* by $x_{n+1}=Tx_n$, for all $n\ge 0$. By non-decreasing property of *T* we get,

2 $$\begin{aligned} x_0\preceq Tx_0 = \ x_1\preceq \ Tx_1 = \ x_2\preceq ... \preceq \ x_n\preceq \ Tx_n = \ x_{n+1}\preceq \ ... \end{aligned}$$

If $x_n=x_{n+1}$ for some $n\ge 0$, then $Tx_n= x_{n+1} = x_n$, so that $x_n$ is a fixed point of *T* and this completes the proof. $\square$

Now assume that $\ x_n\ne x_{n+1}$, for all $\ n\ge 0$. Since $\ T$ is $\alpha$-admissible, we have

$$\begin{aligned} \alpha (x_0,Tx_0 )=\alpha (x_0,x_1 )\ge 1\Rightarrow \alpha (Tx_0,Tx_1 )=\alpha (x_1,x_2 )\ge 1. \end{aligned}$$

Also, we get

$$\begin{aligned} \alpha \ (x_1,x_2)\ge 1\Rightarrow \alpha (Tx_1,Tx_2) =\alpha (x_2,x_3)\ge 1. \end{aligned}$$

By induction we obtain,

3 $$\begin{aligned} \alpha (x_n,x_{n+1} )\ge \ 1, \text { for all } \ n\ge 0. \end{aligned}$$

We consider two cases.

**Case (i):** If $$A=d(x_n,Tx_{n-1})+d(x_{n-1},Tx_n)\ne 0$$.

Now, by applying (1) and (3) for all $n\ge 1$ we get,

$$\begin{aligned} d(x_n,x_{n+1}) &=\, d(Tx_{n-1},Tx_n)\\\le\, & {} \alpha (x_{n-1},x_n)d(Tx_{n-1},Tx_n)\\ &\le\, ad(x_{n-1},x_n)+b[d(x_{n-1},Tx_{n-1})+d(x_{n-1},Tx_n)]\\ &+ c \frac{d(x_n,Tx_n)+d(x_n,Tx_{n-1})}{1+d(x_n,Tx_n)d(x_n,Tx_{n-1})}\\&+e\frac{d(x_{n-1},Tx_{n-1})d(x_{n-1},Tx_n)+d(x_n,Tx_{n-1})d(x_n,Tx_n )}{d(x_n,Tx_{n-1})+d(x_{n-1},Tx_n)}\\&+ f\frac{d(x_n,Tx_n)[1+d(x_{n-1},Tx_{n-1})]}{1+d(x_{n-1},x_n)}\\ &= \,ad(x_{n-1},x_n)+b[d(x_{n-1},x_n)+d(x_{n-1},x_{n+1})] +c \frac{d(x_n,x_{n+1})+d(x_n,x_n)}{1+d(x_n,x_{n+1})d(x_n,x_n)}\\&+e\frac{d(x_{n-1},x_n)d(x_{n-1},x_{n+1}) +d(x_n,x_n )d(x_n,x_{n+1})}{d(x_n,x_n )+d(x_{n-1},x_{n+1})} +f \frac{d(x_n,x_{n+1})[1+d(x_{n-1},x_n)]}{1+d(x_{n-1},x_n)}\\ & \le\, ad(x_{n-1},x_n)+bd(x_{n-1},x_n)+bd(x_{n-1},x_{n+1}) \\&+cd(x_n,x_{n+1})+ed(x_{n-1},x_n)+fd(x_n,x_{n+1}). \end{aligned}$$

That is,

$$\begin{aligned} d(x_n,x_{n+1})&\le {} ad(x_{n-1},x_n)+bd(x_{n-1},x_n) +bsd(x_{n-1},x_n)+bsd(x_n,x_{n+1})\\&+cd(x_n,x_{n+1}) +ed(x_{n-1},x_n)+fd(x_n,x_{n+1}), \end{aligned}$$

which gives $$(1-(bs+c+f))d(x_n,x_{n+1})\le (a+b(1+s)+e)d(x_{n-1},x_n)$$,

4 $$\begin{aligned} d(x_n,x_{n+1})\le \frac{(a+b(1+s)+e)}{1-(bs+c+f)}d(x_{n-1},x_n). \end{aligned}$$

Let $$\frac{(a+b(1+s)+e)}{1-(bs+c+f)} =\beta \in [0,\frac{1}{s})$$.

Now (4) becomes,

$$\begin{aligned} \ d(x_n,x_{n+1})\le\, \beta d(x_{n-1},x_n). \end{aligned}$$

Also, we obtain

$$\begin{aligned} d(x_{n-1},x_n)\le\, \beta d(x_{n-2},x_{n-1}). \end{aligned}$$

So, we have

$$\begin{aligned} d(x_n,x_{n+1})\le \,\beta ^{2} d(x_{n-2},x_{n-1}). \end{aligned}$$

By continuing this process inductively we get,

$$\begin{aligned} d(x_n,x_{n+1})\le\, \beta ^{n} d(x_0,x_1) \text { for all } n\ge 1. \end{aligned}$$

Since $$0\le\, \beta <\frac{1}{s}$$,

$$\begin{aligned} \beta ^{n} d(x_0,x_1)\rightarrow 0, as \ n\rightarrow \infty . \end{aligned}$$

Hence, $d(x_n,x_{n+1})\rightarrow 0$, as $n\rightarrow \infty$. That is,

5 $$\begin{aligned} \lim _{n\rightarrow \infty }d(x_n,x_{n+1})=0. \end{aligned}$$

Now, we show the sequence $\{x_n\}$ is b-Cauchy.

For $m,n\in N$ with $\ m> n$, applying the triangle inequality we get,

$$\begin{aligned} d(x_n,x_m)\le\, & {} s[d(x_n,x_{n+1})+d(x_{n+1},x_m)] \\\le\, & {} sd(x_n,x_{n+1})+s^{2} [d(x_{n+1},x_{n+2}) +\cdots + d(x_{m-1},x_m)] \\\le\, & {} sd(x_n,x_{n+1})+\ s^{2} d(x_{n+1},x_{n+2})+\cdots + s^{m} d(x_{m-1},x_m) \\\le\, & {} s\beta ^{n} d(x_0,x_1)+\ s^{2} \beta ^{n+1} d(x_0,x_1) +\cdots + s^{m} \beta ^{m-1} d(x_0,x_1) \\=\, & {} s\beta ^{n} d(x_0,x_1)[1+(s\beta )+(s\beta )^{2}+ \cdots + (s\beta )^{m-(n+1)}]\\\le \,& {} s\beta ^{n} d(x_0,x_1)[1+(s\beta )+(s\beta )^{2}+ \cdots ]\\= \,& {} \frac{s\beta ^{n}}{1-s\beta } d(x_0,x_1). \end{aligned}$$

Thus,

$$\begin{aligned} d(x_n,x_m )\le \frac{s\beta ^{n}}{1-s\beta }d(x_0,x_1). \end{aligned}$$

Since $\beta \in [0,\frac{1}{s}),$ we get,

$$\begin{aligned} \frac{s\beta ^{n}}{1-s\beta } d(x_0,x_1)\rightarrow 0 \text { as } \ n\rightarrow \infty . \end{aligned}$$

Hence, $d(x_n,x_m)\rightarrow 0$ as $m,n\rightarrow \infty$. Therefore, $\{x_n\}$ is a b-Cauchy sequence in *X*.

Since, *X* is b-complete, there exists $x\in X$ such that $x_n\rightarrow x$, that is,

6 $$\begin{aligned} \lim _{n\rightarrow \infty }{x_n}=x. \end{aligned}$$

Since, *T* is continuous,

7 $$\begin{aligned} x=\lim _{n\rightarrow \infty }{x_{n+1}} = \lim _{n\rightarrow \infty }{Tx_n} = T(\lim _{n\rightarrow \infty }{x_n})=Tx. \end{aligned}$$

That is, $Tx=x$. So, *x* is a fixed point of *T*.

**Case (ii):** If $A=d(x_n,Tx_{n-1})+d(x_{n-1},Tx_n)=0,$ from (1) we have $d(x_n,x_{n+1})=0$, which gives that $x_n=x_{n+1}$, it is a contradiction as the elements of the sequence are comparable and distinct. Therefore, *T* has a fixed point. By removing the continuity assumption of *T* in Theorem 3 we get the following result.

### Theorem 4

*Let* $(X,d,\preceq )$ *be a complete partially ordered* *b*-*metric space with coefficient* $s\ge 1$*,* $\alpha :X\times X\rightarrow R^{+}$ *and* $T:X\rightarrow X$*, satisfies the following conditions:*

(i): *T* *is generalized rational type* $\alpha$-*admissible contractive mapping;*
(ii): *there exists a point* $x_0\in X$ *such that* $x_0\preceq Tx_0$ *and* $\alpha (x_0,Tx_0)\ge 1;$
(iii): *there exists a non-decreasing sequence* ${x_n }\rightarrow x$ *in* *X, with* $x_n\preceq x$ *and* $\alpha (x_{n},x) \ge 1$ *for all* $n \ge 0$*;*
(iv): *T* *is a non-decreasing mapping with regards to* $\preceq$*;*
(v): *d* *is continuous;*
(vi): *T* *is an* $\alpha$-*admissible mapping*.

*Then* *T* *has a fixed point in* *X*.

### Proof

By (*ii*) there exists $x_0\in X$ such that $x_0\preceq Tx_0$ and $\alpha (x_0,Tx_0)\ge 1$.

We define a sequence $\{x_n\}$ in *X* by $T^{n+1}x_0=T(T^{n}x_0)$, for all $n\ge 0$. Since *T* is non-decreasing we have

8 $$\begin{aligned} x_0\preceq Tx_0=x_1\preceq T^{2}x_0=x_2\preceq T^{3}x_0=x_3\preceq \ ... \preceq x_n\preceq T^{n+1}x_0=x_{n+1}\preceq \ ... . \end{aligned}$$

Following as in the proof of Theorem 3 we get that the sequence $\{x_n\}$ is b-Cauchy in *X* and it converges to $x\in X$.

Now, we have to show the existence of a fixed point of *T* in *X*.

That is, $x = Tx$. Suppose that $x \ne Tx$. By (iii), there exists a sequence $\{x_{n}\}$ in *X* such that $\alpha (x_{n}, x) \ge 1$ for all $n \in {\mathbb {N}}\cup \{0\}$. We can suppose that $x_{n} \ne Tx$ for all $n \in {\mathbb {N}}\cup \{0\}$. We consider the following cases.

**Case (i):** If $\ A=d(x,Tx_{n})+d(x_{n},Tx)\ne 0$, then from (1), we have

$$\begin{aligned} d(x_{n+1},Tx)= \,& {} d(Tx_{n},Tx)\\\le & {} \alpha (x_{n}, x)d(Tx_{n},Tx) \\\le\, & {} ad(x_{n},x)+b[d(x_{n},Tx_{n})+d(x_{n},Tx)] +c\frac{d( x,Tx)+d( x,Tx_{n})}{1+d( x, Tx)d( x,Tx_{n})} \\&+\,e\frac{d(x_{n},Tx_{n})d(x_{n},Tx)+d( x,Tx_{n})d( x,T x)}{d( x,Tx_{n})+d(x_{n},T x)}\\&+\,f\frac{d( x,T x)[1+d(x_{n},Tx_{n})]}{1+d(x_{n}, x)}. \end{aligned}$$

So,we have

$$\begin{aligned} d(x_{n+1},Tx)\le\, & {} ad(x_{n},x)+b[d(x_{n},x_{n+1})+d(x_{n},Tx)] +c\frac{d( x,Tx)+d( x,x_{n+1})}{1+d( x, Tx)d( x,x_{n+1})} \\&+\,e\frac{d(x_{n},x_{n+1})d(x_{n},Tx)+d( x,x_{n+1})d( x,T x)}{d( x,x_{n+1})+d(x_{n},T x)}\\&+\,f\frac{d( x,T x)[1+d(x_{n},x_{n+1})]}{1+d(x_{n}, x)}. \end{aligned}$$

Letting limit as $n\rightarrow \infty$ in the above inequality we obtain,

$$\begin{aligned} d(x,Tx)\le & {} bd(x,Tx)+cd(x,Tx)+fd(x,Tx),\\= & {} (b+c+f)d(x,Tx). \end{aligned}$$

It follows that $(1-(b+c+f))d(x,Tx) \le 0.$ Since $(1-(b+c+f))>0$, we must have $d(x,Tx)=0$, that is, $x=Tx$. Hence, *x* is a fixed point of *T*.

**Case (ii)** $A=d(x,Tx_{n})+d(x_{n},Tx)=0$ then from (1), we have

$$\begin{aligned} d(x_{n+1},Tx)=0, \end{aligned}$$

by taking limit as $n\rightarrow \infty$ we get, $d(x,Tx)=0,$ which implies that $x=Tx$.

Therefore, *x* is a fixed point of *T*. $\square$

In the following we use Condition (U) to guarantee the uniqueness of fixed point in Theorem 3 and Theorem 4.

**Condition U:** For all $x,y\in Fix(T)$, we have $\alpha (x,y)\,\ge\, 1$, where *Fix*(*T*) denotes the set of all fixed points of *T*. Every pair of elements has a lower bound and has an upper bound. This condition is equivalent to for every $x,y\in X$ there exists $z\in X$ which is comparable to *x* and *y*.

### Theorem 5

*In addition to the hypotheses of Theorem* 3*(or Theorem* 4*), condition* (U) *provides uniqueness of fixed point of* *T* *in* *X*.

### Proof

From Theorem 3 (or Theorem 4) the set of fixed points of *T* is nonempty. Suppose that *x* and *y* are two fixed points of *T* then, we claim that $x = y$. Suppose that $x\ne y$.

We consider the following cases:

**Case (i)** If $A=d(y,Tx)+d(x,Ty)\ne 0$ then from (1), we have

$$\begin{aligned} d(x,y)\,=\, & {} d(Tx,Ty)\\\le & {} \alpha (x,y)d(Tx,Ty)\\\le\, & {} ad(x,y)+b[d(x,Tx)+d(x,Ty)]+c\frac{d(y,Ty)+d(y,Tx)}{1+d(y,Ty)d(y,Tx)}\\&+\,e\frac{d(x,Tx)d(x,Ty)+d(y,Tx)d(y,Ty)}{d(y,Tx)+d(x,Ty)} +f\frac{d(y,Ty)[1+d(x,Tx)]}{1+d(x,y)}, \\=\, & {} ad(x,y)+b[d(x,x)+d(x,y)]+c\frac{d(y,y)+d(y,x)}{1+d(y,y)d(y,x)}\\&+\,e\frac{d(x,x)d(x,y)+d(y,x)d(y,y)}{d(y,x)+d(x,y)} +f\frac{d(y,y)[1+d(x,x)]}{1+d(x,y)},\\=\, & {} ad(x,y)+bd(x,y)+cd(y,x),\\= & {} (a+b+c)d(x,y)<d(x,y), \text { since } a+b+c<1, \end{aligned}$$

which is a contradiction, thus we get, $d(x,y)=0$, which implies that $x=y$.

Hence, *T* has a unique fixed point.

**Case (ii)** If $\ A=d(y,Tx)+d(x,Ty)=0$, then from (1), we have $d(x,y)=0$ which implies that $x=y.$ Therefore, *T* has a unique fixed point. $\square$

In the following we give corollaries to our main findings.

### Corollary 1

*Let* $(X,d,\preceq )$ *be a complete partially ordered b-metric space with coefficient* $\ s\ge 1$*,* $\alpha :\ X\times \ X\rightarrow \ R^{+}$ *and* $\ T:X\rightarrow X$*, satisfies the following conditions:*

(i): 9 $$\begin{aligned}&\alpha (x,y)d(Tx,Ty)\le {\left\{ \begin{array}{ll} ad(x,y)+b[d(x,Tx)+d(x,Ty)]+e\frac{d(x,Tx)d(x,Ty)+d(y,Tx)d(y,Ty)}{d(y,Tx)+d(x,Ty)}&{}\quad \text {if }A\ne 0 \\ 0&{}\quad \text {if }A=0 \end{array}\right. }\nonumber \\&\quad \text { for all } \ x,y\in X \text { with } \ x\preceq y, \text { where } \ A=d(y,Tx)+d(x,Ty) \end{aligned}$$
  *and* $$a,b,e\in [0,1)$$ *with* $$as+(2s+s^{2})b+es<1$$*;*
(ii): *there exists a point* $x_0\in X$ *such that* $x_0\preceq Tx_0$ *and* $\alpha (x_0,Tx_0)\ge 1$*;*
(iii): *T* *is an* $\alpha$-*admissible mapping;*
(iv): *T* *is continuous and a non-decreasing mapping with regards to* $\preceq$.

*Then* *T* *has a fixed point in* *X*.

### Proof

The result follows by taking $c=f=0$ in Theorem 3. $\square$

### Remark 1

By taking $\alpha (x,y)=1$ for all $x,y\in X$ and $s=1$ in Corollary 1 we get the result of (Seshagiri and Kalyani, 2020) in metric spaces.

### Corollary 2

*Let* $(X,d,\preceq )$ *be a complete partially ordered b-metric space with coefficient* $s\ge 1$*,* $\alpha :\ X\times \ X\rightarrow \ R^{+}$ *and* $T:X\rightarrow X$ *satisfies the following conditions:*

(i): 10 $$\begin{aligned}&\alpha (x,y)d(Tx,Ty)\le\, {\left\{ \begin{array}{ll} ad(x,y)+b[d(x,Tx)+d(x,Ty)] +c\frac{d(y,Ty)+d(y,Tx)}{1+d(y,Ty)d(y,Tx)} \\ \quad +\,e\frac{d(x,Tx)d(x,Ty)+d(y,Tx)d(y,Ty)}{d(y,Tx)+d(x,Ty)}&{}\quad \text {if }A\ne 0 \\ 0&{}\quad \text {if }A=0 \end{array}\right. }\nonumber \\&\quad \text { for all } x,y\in X \text { with } x\preceq y, \text { where } A=d(y,Tx)+d(x,Ty) \end{aligned}$$
  *and* $$\ a,b,c,e\in [0,1)$$ *with* $$as+(2s+s^{2})b+c+es<1$$*;*
(ii): *if there exists a point* $x_0\in X$ *such that* $x_0\preceq Tx_0$ *and* $\alpha (x_0,Tx_0)\ge 1$*;*
(iii): *T* *is an* $\alpha$-*admissible mapping;*
(iv): *T* *is a non-decreasing mapping with regards to* $\preceq$*;*
(v): *there exists a non-decreasing sequence* ${x_n }\rightarrow x$ *in* *X, with* $x_n\preceq x$ *and* $\alpha (x_{n},x) \ge 1$ *for all* $n \in {\mathbb {N}}$.

*Then* *T* *has a fixed point in* *X*.

### Proof

The result follows by taking $f=0$ in Theorem 4. $\square$

### Remark 2

By taking $\alpha (x,y)=1$ for all $x,y\in X$ in Corollary 2 we get another corollary in *b*-metric spaces.

### Corollary 3

*Let* $(X,d,\preceq )$ *be a complete partially ordered b-metric space with coefficient* $s\ge 1$ *and* $T:X\rightarrow X$ *be a non-decreasing mapping with regards to* $\preceq$ *which satisfies the following condition:*

$$\begin{aligned} d(Tx,Ty)\le\, & {} ad(x,y)+b[d(x,Tx)+d(x,Ty)]+c\frac{d(y,Ty)+d(y,Tx)}{1+d(y,Ty)d(y,Tx)}\\&+\,f\frac{d(y,Ty)[1+d(x,Tx)]}{1+d(x,y)} \end{aligned}$$

*for all* $x,y\in X$ *with* $x\preceq y$*, where* $a,b,c,f\in [0,1)$ *with* $as+(2s+s^{2})b+c+f<1$.

*Then* *T* *has a unique fixed point in* *X*.

### Proof

The result follows by taking $\alpha (x,y)=1$ for all $x,y\in X$ and $e=0$ in Theorem 3. $\square$

### Remark 3

By taking $b=c=f=0$ and $s=1$ for all $x,y\in X$ in Corollary 3 we get Banach fixed point theorem in metric spaces.

### Corollary 4

*Let* $(X,d,\preceq )$ *be a complete partially ordered b-metric space with coefficient* $s\ge 1$, $\alpha :\ X\times \ X\rightarrow \ R^{+}$ *and* $T:X\rightarrow X$ *satisfies the following conditions:*

(i): 11 $$\begin{aligned}&\alpha (x,y)d(Tx,Ty)\le\, {\left\{ \begin{array}{ll} ad(x,y)+c\frac{d(y,Ty)+d(y,Tx)}{1+d(y,Ty)d(y,Tx)}+e\frac{d(x,Tx)d(x,Ty) +d(y,Tx)d(y,Ty)}{d(y,Tx)+d(x,Ty)}\\ \quad +\,f\frac{d(y,Ty)[1+d(x,Tx)]}{1+d(x,y)}&{}\quad \text {if }A\ne 0 \\ 0&{}\quad \text {if }A=0 \end{array}\right. }\nonumber \\&\quad \text { for all } \ x,y\in X \text { with } \ x\preceq y \text { where } \ A=d(y,Tx)+d(x,Ty) \end{aligned}$$
  *and where* $a,c,e,f\in [0,1)$ *with* $as+ c+es+f<1$*;*
(ii): *if there exists a point* $x_0\in X$ *such that* $x_0\preceq Tx_0$ *and* $\alpha (x_0,Tx_0)\ge 1$*;*
(iii): *T* *is an* $\alpha$-*admissible mapping;*
(iv): *T* *is a non-decreasing mapping with regards to* $\preceq$*;*
(v): *there exists a non-decreasing sequence* ${x_n }\rightarrow x$ *in* *X, with* $x_n\preceq x$ *and* $\alpha (x_{n},x) \ge 1$ *for all* $n \in {\mathbb {N}}$.

*Then* *T* *has a fixed point in* *X*.

### Proof

The result follows by taking $b=0$ in Theorem 4. $\square$

### Remark 4

By taking $\alpha (x,y)=1$ for all $x,y\in X$ in Corollary 4 we get another corollary in *b*-metric spaces.

## Application to integral equation

In this section we use Corollary 3 to show that there is a solution to the following integral equation,

12 $$\begin{aligned} x(t)= \int _{0}^{1} L(t,r,x(r))dr. \end{aligned}$$

Let $X=C[0,1]$ be the set of real continuous functions defined on [0, 1]. We endow *X* with partial order $\preceq$ given by $x\preceq y$ if and only if $x(t)\preceq y(t)$ for all $t\in [0,1]$ and

$$\begin{aligned} d(x,y)=\max \limits _{t\in [0,1]}(| x(t)-y(t)|)^{m} \end{aligned}$$

for all $x,y \in X$, where $m\ge 1$. It is evident that $(X,d,\preceq )$ is a complete partial ordered b-metric space with a parameter $s=2^{m-1}$. Consider the mapping $T: X\rightarrow X$ given by $Tx(t)=\int _{0}^{1} L(t,r,x(r))dr$.

### Theorem 6

*Consider Equation* (12) *and suppose that*

(i) $L: [0,1]\times [0,1]\times R \rightarrow R^{+}$ *is continuous function;*
(ii) *there is a continuous function* $\beta : [0,1]\times [0,1]\rightarrow R^{+}$ *such that* $\int _{0}^{1} \beta (t,r)dr\le 1$*;*
(iii) *there exists a constant* $a\in [0,1)$ *such that for all* $(t,r) \in [0,1]^{2}$ *and* $x,y \in R$*,*
  $$\begin{aligned} | L(t,r,x(r))- L(t,r,y(r))| \le a^{\frac{1}{m}}\beta (t,r)|x(r)-y(r)|. \end{aligned}$$
  *Then the integral Equation* (12) *has a unique solution* $x\in X$.

### Proof

For $x,y \in X$, from condition (ii) and (iii), for all $t\in [0,1]$, we have

$$\begin{aligned} d(Tx(t),Ty(t))=\, & {} (|Tx(t)- Ty(t)|)^{m}\\= & {} \left( \left| \int _{0}^{1} L(t,r,x(r))dr- \int _{0}^{1} L(t,r,y(r))dr\right| \right) ^{m}\\=\, & {} \left( \left| \int _{0}^{1}( L(t,r,x(r))- L(t,r,y(r)))dr\right| \right) ^{m}\\\le & {} \left( \int _{0}^{1}| L(t,r,x(r))- L(t,r,y(r))|dr\right) ^{m}\\\le & {} \left( \int _{0}^{1}a^{\frac{1}{m}}\beta (t,r)|x(r)- y(r)|dr\right) ^{m}\\=\, & {} \left( \int _{0}^{1}a^{\frac{1}{m}}\beta (t,r)(|x(r)- y(r)|^{m})^{\frac{1}{m}}dr\right) ^{m}\\\le\, & {} \left( \int _{0}^{1}a^{\frac{1}{m}}\beta (t,r)(d(x(t),y(t)))^{\frac{1}{m}}dr\right) ^{m}\\= & {} ad(x,y)\left( \int _{0}^{1}\beta (t,r)dr\right) ^{m}\\\le\, & {} ad(x,y)\\\le & {} ad(x,y)+b[d(x,Tx)+d(x,Ty)]+c\frac{d(y,Ty)+d(y,Tx)}{1+d(y,Ty)d(y,Tx)}\\&+f\frac{d(y,Ty)[1+d(x,Tx)]}{1+d(x,y)}. \end{aligned}$$

Therefore, all conditions of Corollary 3 are satisfied and as a result the mapping *T* has a unique fixed point in *X*. Which is a solution of the integral equation in (12). $\square$

Now, we give an examples to support our main findings.

### Example 3

Let $X=\{1,2,3,4,5\}$. We define *d* as follows:

(i) $$d(x,x)=0,$$ for all $$x\in X$$;
(ii) $d(x,y)=d(y,x),$ for all $x, y\in X$ $d(1,2)=d(2,3)=d(3,4)=d(4,5)=1$
  $$d(1,3)=d(2,4)=d(3,5)=2$$
  $$d(1,4)=d(2,5)=3$$
  $$d(1,5)=m>4$$.

Then $$d(x,y)\le \frac{m}{4}[d(x,z)+d(z,y)]$$, for $$x,y,z\in X$$.

We note that (*X*, *d*) is a *b*-metric space with $s\ge \frac{m}{4}$. But (*X*, *d*) is not a metric space because for $x=1$, $y=5$ and $z=2$, $d(1,5)=m \nleq 4=d(1,2)+d(2,5)$.

Now, we define a partial order on *X* by

$$\begin{aligned} \preceq : = \{(1,1),(2,2),(3,3),(4,4),(5,5),(3,4),(3,5),(4,5)\}. \end{aligned}$$

Then $(X,\preceq )$ is a partially ordered set. We define $T:X\rightarrow X$ and $\alpha :X\times X\rightarrow \Re ^+$ as:

$$\begin{aligned} T(x)= & {} {\left\{ \begin{array}{ll} 1 &{}\quad \text {if }x,y\in \{1,2\}\\ 2 &{}\quad \text {if }x,y\in \{3,4,5\}; \end{array}\right. }\\ \alpha (x,y)= & {} {\left\{ \begin{array}{ll} 1 &{}\quad \text {if }x,y\in \{3,4,5\} \\ 2 &{}\quad otherwise. \end{array}\right. } \end{aligned}$$

Clearly *T* is continuous, non-decreasing and $\alpha$-admissible mapping.

Further for $x_0=1\in X$ we have $\alpha (x_0,Tx_0)=2\ge 1$.

By choosing $s=2$, $a=\frac{1}{32}$, $b=\frac{1}{256}$, $c=e=\frac{1}{64}$ and $e=\frac{1}{128},$ we see that *T* satisfies Inequality (1) and all the hypotheses of the Theorem 3 and *T* has a fixed point $x_0=1$.

### Example 4

Let $X=[0,1]$ be endowed with usual order $\le$ and with the *b*-metric $d:X\times X\rightarrow R^{+}$ defined by $d(x,y)=\frac{1}{16}|x-y|^{2}$ with $\ s = 2$.

We define an operator $\ T: X \rightarrow X$ by

$$\begin{aligned} Tx={\left\{ \begin{array}{ll} \frac{x}{4}&{}\quad \text {if }x\in [0,\frac{1}{4}] \\ \frac{x}{2}+\frac{1}{2}&{}\quad \text {if }x\in (\frac{1}{4},1] \end{array}\right. } \end{aligned}$$

and $$\alpha :X\times X\rightarrow R^{+}$$ by

$$\begin{aligned} \alpha (x,y)={\left\{ \begin{array}{ll} 2&{}\quad \text {if }x,y\in [0,\frac{1}{4}] \\ 1&{}\quad \text {if }x,y\in (\frac{1}{4},1] \\ 0&{}\quad \text {otherwise}. \end{array}\right. } \end{aligned}$$

Clearly *T* is non-decreasing and an $\alpha$-admissible mapping.

Further, for $x_0=0$ we have $\alpha (x_0,Tx_0)=\alpha (0,T0)=2\ge 1$.

Now we verify Inequality (1) with $s=2$ and $a=f=\frac{1}{64}$, $b=\frac{1}{128}$, $c=\frac{1}{32}$ and $e=\frac{1}{64}$ with $\ as+(2s+s^{2})b+c+es+f\ <1$.

**Case (i):** If $\ x,y\in [0,\frac{1}{4}]$ we get,

$$\begin{aligned} \alpha (x,y)d(Tx,Ty)= & {} 2\left( \frac{1}{16}\right) \left| \frac{x}{4}-\frac{y}{4}\right| ^{2}=2\left( \frac{1}{256}\right) |x-y|^{2}=\frac{1}{128}|x-y|^{2}\\\le & {} \frac{1}{64}|x-y|^{2}+\frac{1}{128}\left[ \left| x-\frac{x}{4}\right| ^{2}+\left| x-\frac{y}{4}\right| ^{2}\right] +\frac{1}{32}\frac{\left| y-\frac{y}{4}\right| ^{2}+\left| y-\frac{x}{4}\right| ^{2}}{1+\left| y-\frac{y}{4}\right| ^{2}\left| y-\frac{x}{4}\right| ^{2}}\\&+ \frac{1}{64}\frac{\left| x-\frac{x}{4}\right| ^{2}\left| x-\frac{y}{4}\right| ^{2}+\left| y-\frac{x}{4}\right| ^{2}\left| y-\frac{y}{4}\right| ^{2}}{\left| y-\frac{x}{4}\right| ^{2}+\left| x-\frac{y}{4}\right| ^{2}} +\frac{1}{64}\frac{\left| y-\frac{y}{4}\right| ^{2}\left[ 1+\left| x-\frac{x}{4}\right| ^{2}\right] }{1+|x-y|^{2}}. \end{aligned}$$

**Case (ii):** If $x,y\in (\frac{1}{4},1]$ we get,

$$\begin{aligned} \alpha (x,y)d(Tx,Ty)= & {} 1\left( \frac{1}{16}\right) \left| \frac{x}{2}+\frac{1}{2}-\left( \frac{y}{2}+\frac{1}{2}\right) \right| ^{2}=\frac{1}{16}\left| \frac{x}{2}-\frac{y}{2}\right| ^{2}=\frac{1}{64}|x-y|^{2}\\\le & {} \frac{1}{64}|x-y|^{2}+\frac{1}{128}\left[ \left| x-\frac{x}{4}\right| ^{2}+\left| x-\frac{y}{4}\right| ^{2}\right] +\frac{1}{32}\frac{\left| y-\frac{y}{4}\right| ^{2}+\left| y-\frac{x}{4}\right| ^{2}}{1+\left| y-\frac{y}{4}\right| ^{2}\left| y-\frac{x}{4}\right| ^{2}}\\&+ \frac{1}{64}\frac{\left| x-\frac{x}{4}\right| ^{2}\left| x-\frac{y}{4}\right| ^{2}+\left| y-\frac{x}{4}\right| ^{2}\left| y-\frac{y}{4}\right| ^{2}}{\left| y-\frac{x}{4}\right| ^{2}+\left| x-\frac{y}{4}\right| ^{2}} +\frac{1}{64}\frac{\left| y-\frac{y}{4}\right| ^{2}\left[ 1+\left| x-\frac{x}{4}\right| ^{2}\right] }{1+|x-y|^{2}}. \end{aligned}$$

**Case (iii):** If $y\in [0,\frac{1}{4}]$ and $x\in (\frac{1}{4},1]$, then Inequality (1) trivially holds.

From the Case (i) - Case(iii) considered above, *T* satisfies Inequality (1) and hence, *T* satisfies all the hypotheses of the Theorem 4 and $0,1\in X$ are fixed points of *T*.

## Limitations

Seshagiri and Kalyani [1] established fixed point results for mappings satisfying certain rational type contractive conditions in complete partial ordered metric spaces and proved the existence and uniqueness of fixed points. In this paper, we define generalized rational type $\alpha$-admissible contractive mappings in the setting of complete partially ordered *b*-metric spaces and prove the existence and uniqueness of fixed points for the introduced mappings. Our results extend and generalize the work of Seshagiri and Kalyani [1] from metric space to *b*-metric space. We have also supported the main results of this paper by applicable examples.
